# Adverse Event Profiles of Adalimumab in Children: A Disproportionality Analysis

**DOI:** 10.3390/ph17081028

**Published:** 2024-08-05

**Authors:** Wenting Zhang, Ziqi Xu, Yamin Shu, Sainan Shu, Qilin Zhang

**Affiliations:** 1 Department of Pharmacy, Tongji Hospital, Tongji Medical College, Huazhong University of Science and Technology, Wuhan 430030, China; wenting@hust.edu.cn (W.Z.); shuyamin1990hust@163.com (Y.S.); 2 Department of Clinical Pharmacy, General Hospital of Central Theater Command of Chinese People’s Liberation Army, Wuhan 430060, China; chinaziqi@163.com; 3 Department of Pediatric Gastroenterology and Infection, Tongji Hospital, Tongji Medical College, Huazhong University of Science and Technology, No. 1095 Jiefang Avenue, Wuhan 430030, China; 4 Department of Pharmacy, Union Hospital, Tongji Medical College, Huazhong University of Science and Technology, No. 1277 Jiefang Avenue, Wuhan 430022, China

**Keywords:** adalimumab, adverse event, children, disproportionality analysis, FAERS database

## Abstract

Background: Adalimumab has been approved by the U.S. Food and Drug Administration (FDA) for the treatment of adult rheumatoid arthritis (RA), and subsequently approved for pediatric treatment of various autoimmune diseases in children of different ages. Due to genetic differences between children and adults in terms of physiology and immunity, there is a need to explore the safety of adalimumab in children in the real world. The aim of this study is to identify potential adverse event (AE) signals associated with the use of adalimumab in pediatric patients (<18 years old) using data from the FDA Adverse Event Reporting System (FAERS). Methods: AEs associated with adalimumab in pediatric patients reported in the FAERS database from the first quarter (Q1) of 2017 to the third quarter (Q3) of 2022 were systematically gathered. Reporting odds ratio (ROR), the proportional reporting ratio (PRR), the information component (IC), and the empirical Bayes geometric mean (EBGM) were used to assess the relationship between adalimumab and AEs in children. Results: Out of 8,363,304 reports collected from the FAERS database during the study period, 3819 reports on children on adalimumab were identified. Adalimumab-related AEs reports were concentrated on 10 toxicity areas and a total of 202 positive signals were detected, of which injection site papule (ROR = 261.97) and intestinal fistula (ROR = 122.09) had the strongest signals. Unexpected significant AEs, including intestinal obstruction, immunodeficiency, abdominal abscess, and Takayasu’s arteritis might also occur. In comparison with patients of all ages in the same time window, the median onset time of children was shorter (99 vs. 149 days). Most of the AE cases occurred in children within the first 1 (1.71%), 2 (8.12%), and 3 months (8.39%) and had early failure types after adalimumab initiation. Methotrexate, folic acid, prednisone, azathioprine, and mesalamine were the top five drugs used concomitantly for adalimumab-associated AEs. Conclusions: When adalimumab is used in children, especially in the first 3 months of treatment, in addition to the AEs recorded in the drug package insert, close attention should be paid to the new potential AEs off-label to ensure the safety of adalimumab in children.

## 1. Introduction

Adalimumab is a fully human monoclonal antibody that specifically binds to tumor necrosis factor (TNF) α and blocks its interaction with TNF receptors on the surface of p55 and p75 cells [1]. TNF is a naturally occurring cytokine involved in normal inflammatory and immune responses, and its abnormal production is associated with many chronic immune inflammatory diseases. Since 2002, adalimumab has been approved by the USA Food and Drug Administration (FDA) for the treatment of adult rheumatoid arthritis (RA), and subsequently approved for pediatric treatment of various autoimmune diseases in children of different ages, such as non-infectious uveitis (NIU), polyarticular juvenile idiopathic arthritis (JIA), plaque psoriasis (PP), ulcerative colitis (UC), Crohn’s disease (CD), and hidradenitis suppurativa [2]. Due to the lack of murine variable domain of immunoglobulin Fab, adalimumab is less immunogenic than infliximab [3]. However, several adverse events (AEs), including thrombocytopenia, leukopenia, recurrence of malignancy, tuberculosis, and cardiac toxicity, occurred in patients treated with adalimumab [4,5]. For the treatment of pediatric CD with adalimumab, about half of the patients reported AEs, the most common being infection, followed by injection-site adverse reactions, joint pain, and muscle pain [6]. The incidence of serious AEs was 11.5%, among which hematologic AEs were the most common, and 35.2% of the cases required drug discontinuation [7].

Although the types and incidence of adverse reactions after using adalimumab in children are similar to those in adults, children are genetically different from adults in terms of physiology and immunity. Furthermore, The safety of adalimumab in pediatric patients has only been evaluated in a small sample, such as the clinical trials or case reports [8]. Given the recognized limitations and observed methodological biases of a single clinical trial in evaluating the safety of adalimumab, especially in detecting rare, uncommon, and delayed AEs, it is of paramount importance to conduct a comprehensive, systematic pharmacovigilance study on adalimumab in such special populations based on a real-world large-sample database. This study retrospectively analyzed the relevant data in the FDA adverse event reporting system (FAERS) database to mining the signals of suspected AEs associated with adalimumab in children, to find the characteristics of potential risk signals in children and provide a basis for the safe use of adalimumab in pediatric clinical practice.

## 2. Results

### 2.1. General Characteristics

During the study period, a total of 8,363,304 reports were documented in the FAERS database after extracting and cleaning data, among which 3819 reports on children on adalimumab were identified. After reviewing those reports, 3759 of them were from adalimumab originators, while the remaining 60 were from adalimumab biosimilars. The characteristics of the included cases are presented in Table 1 and Table S1. Most cases were focused on the period from 2017 to 2022, reflecting the increasing clinical use of adalimumab in children in recent years. The proportion of females (51.27%) was slightly higher than that of males (45.48%). Most cases are between 13 and 18 years (55.33%), with a median age of 14 (IQR 11–16) years. Most of reports came from the US (58.31%) and mainly submitted by consumers (58.55%). Crohn’s disease was the most reported indication (43.18%), followed by juvenile idiopathic arthritis (20.19%), colitis ulcerative (5.94%), uveitis (3.19%), and hidradenitis (3.09%). Most AE reports were serious outcomes (62.90%), namely, resulting in hospitalization (54.58%), life-threatening (2.16%), or death (1.87%). Methotrexate, folic acid, prednisone, azathioprine, and mesalamine were the top five drugs used concomitantly for adalimumab-associated AEs, with 438 (11.47%), 208 (5.45%), 193 (5.05%), 164 (4.29%) and 109 (2.85%) cases, respectively.

### 2.2. Disproportionality Analysis

Signal strengths and reports of adalimumab at the System Organ Class (SOC) level are shown in Table 2 using a disproportionality method. After excluding SOCs unrelated to the drug, adalimumab-induced AEs reports were concentrated on 10 toxicity areas that at least one of the four algorithms meet the criteria for: general disorders and administration site conditions (n = 1780), gastrointestinal disorders (n = 1609), immune system disorders (n = 856), vascular disorders (n = 855), infections and infestations (n = 648), musculoskeletal and connective tissue disorders (n = 600), eye disorders (n = 306), neoplasms benign, malignant, and unspecified (n = 152), and ear and labyrinth disorders (n = 54).

Results showed that adalimumab involved a total of 202 positive signals after conforming to all of the four algorithms simultaneously. The number of reporting PTs ≥ 10 are summarized in Table 3, including 112 PTs of which injection site papule (ROR = 261.97) and intestinal fistula (ROR = 122.09) had the strongest signals, and others are listed in Supplementary Table S2. In our data analysis, PTs of cataract, visual impairment, injection site pain, injection site hemorrhage, abdominal pain, gastrointestinal inflammation, white blood cell counts increased, disseminated tuberculosis, arthritis, systemic lupus erythematosus, synovitis, lymphoma, and depressed mood presented significant signals, which were consistent with the instructions and medication warnings. However, hypertension, arrhythmology, myocardial infarction, hypercholesterolemia, dehydration, hypokalemia, pneumonia, upper respiratory tract infection, urinary and genital tract infections, headache, alanine aminotransferase increased, hepatic steatosis, tinnitus and otitis media, etc., which were listed on the drug label, did not show positive signals. It was noteworthy that unexpected significant AEs, including intestinal obstruction, immunodeficiency, abdominal abscess, and Takayasu’s arteritis, were uncovered in the label.

### 2.3. Time to Onset of Adalimumab-Related Adverse Events

**Figure 1:**
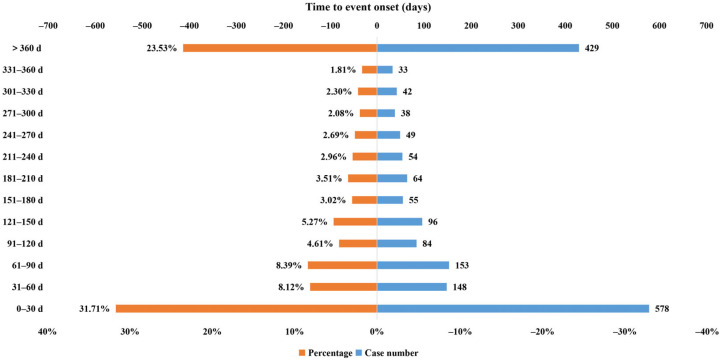
Time to onset of adalimumab-related AEs in children (<18 years).

**Figure 2:**
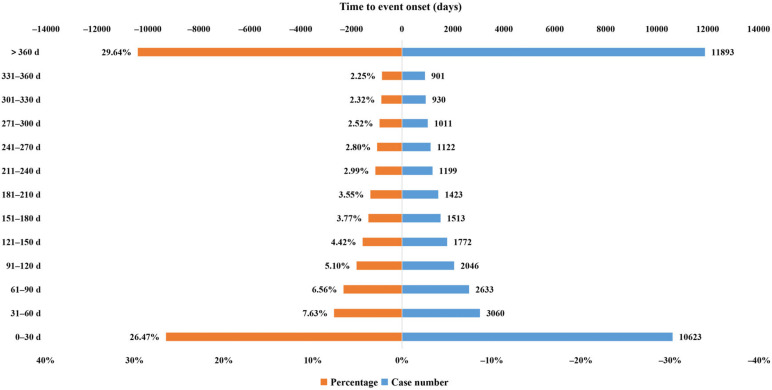
Time to onset of adalimumab-related AEs in all ages.

Results in Table 4 indicated that a total of 1823 adalimumab-associated cases in children reported onset time after excluding unreported or unknown onset time reports. Within the timeframe of this study, the median onset time of children was 99 (14–336) days, which is shorter compared to adults with an onset time of 151 (28–440) days, resulting in a combined median onset time of 149 (28–434) days across all cases. Most of the AE cases occurred in children within the first 1 (n = 578, 31.71%), 2 (n = 148, 8.12%), and 3 months (n = 153, 8.39%) after adalimumab initiation (Figure 1), which is basically consistent with the onset time of AEs in the whole cases of the database (Figure 2). Of note, the AEs might still occur after 1 year of adalimumab treatment with the proportion of 23.53% in children’s cases. WSP analysis demonstrated that the upper limits of 95% CI of the shape parameters β were less than 1, indicating that these AEs had early failure types and the risk of AEs occurrence gradually decreased over time.

## 3. Discussion

The most frequently reported adverse effects that have been documented on the label of adalimumab include infections (e.g., nasopharyngitis, upper respiratory tract infection, and sinusitis), injectable site reactions (erythema, pruritus, bleeding, pain, or swelling), headache, and musculoskeletal pain. Serious AEs included fatal and life-threatening infections, a variety of malignant tumors, and severe hematological reactions. These AEs are highly consistent with the signals mined by FAERS in this study. There was no significant difference in reported AEs between adults and children. The statistically significant SOCs associated with adalimumab involved general disorders and administration site conditions, gastrointestinal disorders, immune system disorders, vascular disorders, infections and infestations, musculoskeletal and connective tissue disorders, eye disorders, neoplasms benign, malignant, and unspecified (including cysts and polyps), and ear and labyrinth disorders, corresponding to the findings of WHO-VigiAccess on investigating the adverse effects of anti-TNFα drugs [9], and also similar to the results of a systematic review on evaluating the efficacy and safety of biologic agents in the treatment of moderate-to-severe Crohn’s disease [10], which confirms the reliability of this study to some extent.

According to the disproportionality analysis, results indicated that the most commonly reported and significant signals at the SOC level were general disorders and administration site conditions, containing the largest number of AE reports (n = 1780). At the PT level, injection site pain (n = 288), injection site hemorrhage (n = 271), pain (n = 120), and injection site erythema (n = 77) were the most reported AEs. Injection site papule (ROR = 261.97), injection site indentation (ROR = 97.72), injection site injury (ROR = 61.71), and injection site hemorrhage (ROR = 46.15) had the strongest signals. A pharmacovigilance analysis of injection site reactions associated with the use of subcutaneously administered biologic products demonstrated that the most common types of injection site reactions reported by adalimumab were pain (23.3%), irritation (14.1%), and erythema (9.6%), which is consistent with our findings in children [11]. Children or guardians are often instructed by physicians to inject adalimumab at home, which may be the reason for more adverse reactions at the injection site, suggesting that guardians should have relevant professional training before helping injections. Younger age was the only factor reported to be associated with the perception of pain at the injection site, and the perception of subcutaneous administration-related pain in IBD patients has a negative impact on treatment adherence [12]. Some tips such as choosing the right injection site (the front of the thigh or lower abdomen) and different injection sites for each time, avoiding areas with scars, and properly cleaning the injection site need to be followed to reduce adverse reactions and improve compliance.

Among the PTs reported in this study, there are some PTs consistent with the indications, such as uveitis, CD, UC, JIA, and RA showing strong signals, which may be because disease is not effectively controlled or progressed because of poor efficacy rather than adalimumab. Unexpected and significant safety signals requiring clinical attention such as intestinal obstruction, glaucoma, immunodeficiency, abdominal abscess, and Takayasu’s arteritis (TA) were detected in our analysis. TA is a rare granulomatous arteritis that affects the aorta and its major branches. The pathophysiology of TA is poorly understood and is thought to be mediated by a cell-mediated autoimmune disease process [13]. The main causes of secondary TA in children are infectious causes such as parvovirus B19, hepatitis B and C, human immunodeficiency virus (HIV), varicella, rickettsia, bacteria, fungi, and mycobacteria. Medications including some antibiotics, TNF inhibitors, and antithyroid agents and systemic diseases such as systemic lupus erythematosus, juvenile dermatomyositis, juvenile idiopathic arthritis, sarcoidosis, inflammatory bowel disease, and malignancy are also major contributors to TA [14].

The plasma level of TNFα is higher in TA patients. Thus, numerous case series and observational studies support the use of TNF inhibitors for the treatment and sustained remission of patients with severe and refractory TA [15,16]. Some authors have reported cases of TA following treatment with TNFα; blocking TNFα may contribute to vasculitis in patients whose immune homeostasis is already out of balance [17,18,19]. The mechanism of this paradoxical side effect is unclear, and the role of TNFα in the development and progression of vasculitis cannot be determined. The phenomenon might be mediated by the deposition of immune complex (ICs) containing drugs, endogenous immunoglobulin, and/or complement components on the blood vessel wall in a disordered immune homeostasis [17]. Through the toxicity study of therapeutic monoclonal antibodies, adverse reactions such as vascular inflammation have also been observed in experimental animals (including monkeys). Using histopathology and immunohistochemistry (IHC) methods, ICs were observed to be deposited in tissues such as blood vessels, synovium, lung, liver, skin, eye, choroid plexus, or glomerulus or bound to neutrophils, monocytes/macrophages, or platelets. Deposition of ICs can activate complement, kinin, and/or coagulation/fibrinolytic pathways, leading to systemic proinflammatory responses [20]. This can also explain the occurrence of adverse reactions such as general disorders and administration site conditions injury, immune system disorders, vascular disorders, and eye disorders, as well as the abnormalities of related test indicators, such as C-reactive protein increased, inflammatory marker increased, and white blood cell count increased.

ICs clearance is biphasic in the human body, with initial transfer from plasma to the liver and/or spleen and subsequent clearance, and the blood complement receptor 1 (CR1) or CR1-like erythrocytes are the key to the transfer process [21,22]. About 85% of the CR1 in human blood are on erythrocytes. In our study, adalimumab was mainly used for Crohn’s disease and juvenile idiopathic arthritis in children, accounting for 43.18% and 20.19%, respectively. According to the commonly used dose of adalimumab, the dose per unit body weight of children was higher than that of adults. The blood volume of children is usually less than that of adults, and the small number of erythrocytes leads to the slow clearance of ICs containing drugs in children, which may be the reason for TA in children.

Methotrexate, folic acid, prednisone, azathioprine, and mesalamine were the top five drugs used concomitantly for adalimumab-associated AEs. The main mechanism of action of methotrexate is competitive inhibition of folate reductase, which has a broad spectrum of antitumor activity. The most common adverse reactions are bone marrow suppression and mucosal injury, usually in combination with folic acid as a supplement. Prednisone has strong anti-inflammatory and appropriate immunosuppressive effects, and azathioprine is usually used concomitantly with prednisone for a variety of autoimmune diseases. Long-term immunosuppression caused by the combination of azathioprine increases the risk of serious infections and malignancies in humans. A recent systematic review of a meta-analysis of 261,698 patients found that both anti-TNFα drugs and thiopurine were associated with an increased risk of lymphoma [23]. A meta-analysis of data from randomized controlled trials (RCTs) of CD in patients treated with adalimumab indicated that combination therapy was associated with an increased risk of non-melanoma skin cancer and other types of malignancies [24]. Patients with defects in the TMPT and NUDT15 genes need to be closely monitored for the risk of myelosuppression caused by azathioprine [25]. Adverse effects of mesalazine are relatively rare and can cause mild stomach discomfort. Taken together, these drugs can lead to an increased risk of immunosuppression, mucosal injury, bone marrow suppression, and malignancies, which are consistent with many of the PTs we found, suggesting that drug combinations are also of concern in the treatment of adalimumab.

In comparison with cases of all ages, the median onset time of children was shorter (99 vs. 149 days). Moreover, the median onset time of children was shorter than adults (99 vs. 151 days). The possible reason is that children’s immune system, liver, and kidney function are still in the development stage, and the function is less mature than that in adults. Data from a population pharmacokinetic analysis of 1300 patients with rheumatoid arthritis suggested that the clearance of adalimumab increased with weight gain [1], and children were generally lighter than adults, which might account for the earlier onset of AEs in children. It is recommended that children should be closely observed when using adalimumab, especially in the first three months of administration, and timely detection and corresponding treatment measures should be taken. Our data represent a gradual reduction in the risk of AE occurrence over time. Of note, the AEs might still occur after 1 year of adalimumab treatment with the proportion of 23.53% in children.

We assessed the serious outcomes of adalimumab-associated AEs, including death, life-threatening, hospitalization, disability, and other serious outcomes. Of these, 62.9% were serious outcomes, with 45 deaths. Although total serious outcomes may exceed the total number of reported cases because some cases list more than one serious outcome, the proportion of serious outcomes is much higher than that of nonserious outcomes. Consistently, Maity T et al. [26] investigated 33 RCTs about adalimumab for the treatment of rheumatoid arthritis, Crohn’s disease, and psoriasis in all age groups, which identified 2852 cases of serious adverse drug reactions (ADRs) from 2014 to 2018, accounting for 16.3% of the total number of ADR cases, including seven deaths. Avedillo-Salas et al. [10] found that adalimumab resulted in a serious adverse effect rate of 6.4% in RCTs, representing 10.5% of all adverse effects. Moreover, recent publications have shown an increased prevalence of cutaneous T-cell lymphoma and tuberculosis in patients undergoing TNFα inhibitor therapy [27,28]. A nationwide population-based retrospective cohort analysis suggested that the use of anti-TNFα drugs is associated with an approximately 4 to 8-fold increased risk of active tuberculosis [28]. Through FAERS data mining, we found 13 cases of lymphoma and 11 cases of disseminated tuberculosis in children who were treated with adalimumab. The FDA specifically warns in the drug label that use of adalimumab can increase the risk of serious infections, including tuberculosis, invasive fungal infections, and other opportunistic infections. In addition, lymphoma and other malignancies have been reported in children and adolescents. With the long-term use of adalimumab, immunogenicity was often observed and typically resulted in neutralization and/or clearance of the therapeutic mAb. In some cases, the neutralizing effect could be overcome by increasing the dose or frequency, which is also an important cause of adverse reactions [29]. Post-marketing real-world data will help clinicians focus on serious AEs in children using adalimumab so that timely measures can be taken in the clinic.

There are some limitations inherently in our study. First, multiple unmeasured confounders, such as dose, course of treatment, potential drug–drug interactions, and comorbidities, which may influence the AEs, were not included in the data analysis, leading to inevitable and unquantifiable biases. Second, 58.31% of the reports were submitted by the United States, which is not representative of AEs in Asian children. Third, the safety report did not provide details on patient exposure to the drug without AEs. Thus, we were unable to calculate the incidence of AEs and establish causality, but only provided an estimation of the signal strength, which was only statistically significant. Despite the above limitations, we systematically and comprehensively revealed AEs in children with adalimumab through an extensive analysis of a large real-world sample FAERS database, which provides valuable evidence for healthcare professionals to reduce the risk of adalimumab-associated AEs.

## 4. Methods

### 4.1. Study Design and Data Source

A retrospective, observational pharmacovigilance study was performed to assess the association of AEs as related to adalimumab used in children’s cases that were reported in the FAERS database. The population spectrum in this study was children, and it measured the occurrence of target AEs associated with adalimumab compared to any other drug used in children in the entire database. Based on the date of adalimumab approval and updated data from the database, we inquired data from the first quarter (Q1) of 2017 to the third quarter (Q3) of 2022. The case information in the database spread regularly over seven types of datasets [30].

### 4.2. Procedures

The data during the period above were downloaded from the FAERS official website (https://fis.fda.gov/extensions/FPD-QDE-FAERS/FPD-QDE-FAERS.html, accessed on 3 February 2023). To filter duplicate reports, only the latest case version (the highest primary ID) of every report was retained [31]. Moreover, FDA or manufacturers may delete cases for various reasons including combining cases (Deleted file), and the deleted cases listed quarterly by FDA were further removed in this study [32]. We then screened for reports in the database (DRUG file) containing any one of the following fields in generic or brand names: adalimumab, abrilada, amjevita, cyltezo, hadlima, hulio, humira, hyrimoz, yusimry. The details of the drug names are shown in Supplementary Table S3.

**Figure 3:**
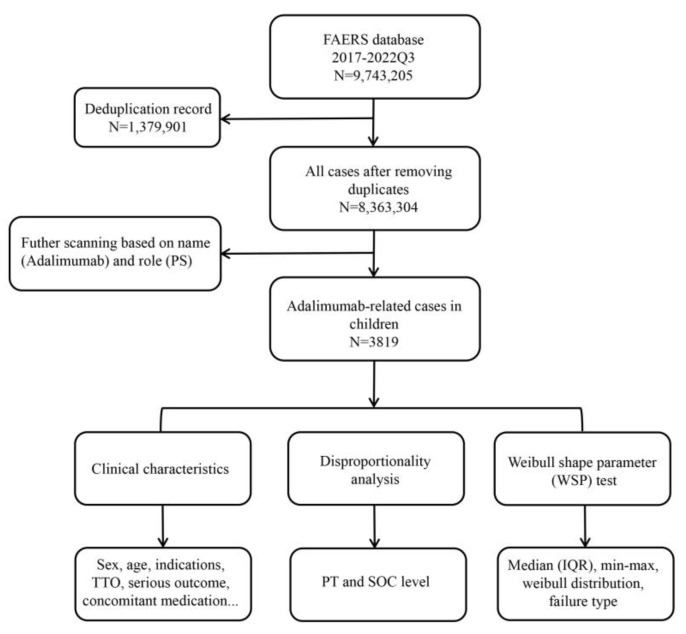
The flow diagram of selecting adalimumab-related AEs in children from FAERS database.

All AEs in the FAERS are coded by the preferred term level of the Medical Dictionary for Regulatory Activities (MedDRA). We performed statistical analysis at the preferred term (PT) level and system organ class (SOC) level in the structure of MedDRA 25.0 terminology. Due to the characteristic of “multi-axiality” in MedDRA terminology, a PT is allowed to be represented in more than one SOC [33]. Accordingly, we classified AEs in each report to the corresponding SOC levels based on MedDRA 25.0. Clinical characteristics of each report were collected, if the data were available, including demographic information (gender, age, weight, reported countries, and reporting year), drug administration details (indication and concomitant medication), date of AE occurrence (time-to-onset), and its outcomes. The cases were serious outcomes if at least one of the following outcomes were reported: death, life-threatening, hospitalization, disability, or other serious medical event [34]. The details of the multi-step process of data extraction, processing, and analysis are shown in Figure 3. All data processing was performed using MYSQL 8.0 (Oracle, Redwood, CA, USA), Microsoft EXCEL 2019, and the GraphPad Prism 8 (GraphPad Software, San Diego, CA, USA).

### 4.3. Statistical Analysis

A disproportionality analysis was employed to compare the proportion of occurring AEs between adalimumab used in children (cases) and all other drugs used in children (non-cases), also known as case/non-case analysis [35]. Both Frequentist and Bayesian methods based on the principles of calculations using a 2 × 2 table, including the reporting odds ratio (ROR), the proportional reporting ratio (PRR), the information component (IC), and the empirical Bayes geometric mean (EBGM), were performed to investigate the association between adalimumab and AEs in this study [30]. These methods are well-validated measures for detecting disproportionate reporting signals in the post-marketing passive monitoring database. To ensure the stability of the detected signals, the AE was considered a statistically significant signal when all the disproportionality measures (ROR, PRR, IC, and EBGM) have crossed the significance threshold. The formulas and thresholds of the four algorithms are shown in Supplementary Table S4. Due to the limitations of spontaneous reporting system data and the different conditions and principles that restrict signal generation in various algorithms, there are differences in the number of signals generated and false positive results [36]. For example, ROR can estimate relative risk to reduce bias; PRR has lower specificity, making it prone to detecting false positive signals but with high sensitivity; MGPS is currently the only method that uses stratified analysis, which is less affected by confounding factors such as demographic data and allows for associations between drug use characteristics and AEs. When there are few reports, EBGM is more stable than PRR. IC has lower quality requirements for AE reporting data but can detect strong correlations in AEs. Therefore, we adopt a combination of multiple methods to reduce bias and minimize false positives.

Time-to-onset (TTO) is defined from the start of adalimumab use to the occurrence of the AE [37]. After removing the invalid data, we performed the median (interquartile range, IQR), min-max, and the Weibull shape parameter (WSP) test separately, to analyze TTO data of adalimumab in the children group and the all group. WSP was used to predict the hazard of the occurrence of AE over time, and the selection of shape parameter and differentiation criterion were described in previous studies [38,39,40]. All statistical analysis and TTO data analysis were performed in Microsoft EXCEL 2019 and Minitab statistical software (v20.0; Minitab LLC, State College, PA, USA).

## 5. Conclusions

Based on the FAERS database, we evaluated the post-marketing safety profiles of adalimumab in children. Adalimumab-related AEs reports were concentrated on 10 toxicity areas, and a total of 202 positive signals were detected. It was noteworthy that unexpected significant AEs, including intestinal obstruction, immunodeficiency, abdominal abscess, and Takayasu’s arteritis, might also occur. The median onset time of children was 99 (IQR 14–336) days and had early failure types, suggesting the risk of AEs occurrence gradually decreased over time. This study can provide important evidence support for further research and clinical practice of adalimumab in children.

## Figures and Tables

**Table 1:** Clinical characteristics of reports with adalimumab (in children) from the FAERS database (January 2017 to September 2022).

Characteristics	Adalimumab-Induced AEs Reports (n = 3819)		
Number of Events	Available Number, n	Case Number, n	Case Proportion, %
Gender, n (%)	3695	-	96.75
Female	-	1958	51.27
Male	-	1737	45.48
Missing	-	124	3.25
Age (years), n (%)	3819	-	100.00
0 < 7	-	461	12.07
7≤ and ≤13	-	1245	32.60
13< and <18	-	2113	55.33
Median (IQR)	-	14 (11–16)	-
Weight (Kg), n (%)	1161	-	30.40
<20	-	114	2.99
20≤ and ≤50	-	487	12.75
>50	-	560	14.66
Median (IQR)	-	49.94 (35–61.29)	-
Missing	-	2658	69.60
Reported countries, n (%)	3621	-	94.82
US	-	2227	58.31
Non-US	-	1394	36.50
Missing	-	198	5.18
Indications, n (%)	3549	-	92.93
Crohn’s disease	-	1649	43.18
Juvenile idiopathic arthritis	-	771	20.19
Colitis ulcerative	-	227	5.94
Uveitis	-	122	3.19
Hidradenitis	-	118	3.09
Missing	-	270	7.07
Outcomes, n (%)	3819	-	100.00
Non-serious outcome	-	1417	37.10
Serious outcome ^a^	-	2402	62.90
Death	-	45	1.87
Life-threatening	-	52	2.16
Hospitalization	-	1311	54.58
Disability	-	60	2.50
Other serious outcomes	-	1474	61.37
Time-to-onset (days)	1823	-	47.74
Median (IQR)	-	99 (14–336)	-
Missing	-	1996	52.26
Reporters, n (%)	3751	-	98.22
Health professional	-	1515	39.67
Consumer	-	2236	58.55
Missing	-	68	1.78
Concomitant medication (Top 5)	1389	-	36.37
Methotrexate	-	438	11.47
Folic acid	-	208	5.45
Prednisone	-	193	5.05
Azathioprine	-	164	4.29
Mesalamine	-	109	2.85
Missing	-	2430	63.63
Reporting year, n (%)	3819	-	100.00
2022 Q3 ^b^	-	627	16.42
2021	-	718	18.80
2020	-	728	19.06
2019	-	672	17.60
2018	-	553	14.48
2017	-	521	13.64

^a^, Total serious outcomes may exceed the total number of reported cases because some cases list more than one serious outcomes. ^b^, The third quarter of 2022. IQR, interquartile range.

**Table 2:** Signal strength of reports of adalimumab (in children) at the System Organ Class (SOC) level in FAERS database.

System Organ Class (SOC)	Adalimumab Cases Reporting SOC	ROR (95% Two-Sided CI)	PRR (χ^2^)	IC (IC025)	EBGM (EBGM05)
General disorders and administration site conditions	1780	1.71 (1.61–1.83) *	1.38 (278.36)	0.46 (0.38) *	1.37 (1.29)
Injury, poisoning, and procedural complications	1609	1.02 (0.96–1.09)	1.01 (0.49)	0.02 (−0.06)	1.01 (0.95)
Gastrointestinal disorders	1197	2.66 (2.48–2.85) *	2.14 (827.17) *	1.07 (0.98) *	2.11 (1.96)
Immune system disorders	856	2.27 (2.10–2.45) *	1.98 (457.88)	0.96 (0.86) *	1.96 (1.81)
Vascular disorders	855	2.03 (1.88–2.19) *	1.80 (337.24)	0.83 (0.72) *	1.78 (1.65)
Skin and subcutaneous tissue disorders	788	0.82 (0.76–0.89)	0.86 (24.63)	−0.22 (−0.33)	0.86 (0.79)
Infections and infestations	648	1.38 (1.27–1.51) *	1.32 (56.11)	0.39 (0.27) *	1.31 (1.21)
Nervous system disorders	642	0.67 (0.61–0.73)	0.72 (87.90)	−0.46 (−0.59)	0.73 (0.67)
Musculoskeletal and connective tissue disorders	600	2.34 (2.14–2.56) *	2.13 (378.00) *	1.06 (0.94) *	2.10 (1.92)
Investigations	548	1.47 (1.34–1.61) *	1.40 (69.52)	0.48 (0.35) *	1.40 (1.27)
Respiratory, thoracic, and mediastinal disorders	492	0.86(0.79–0.95)	0.88 (8.94)	−0.18 (−0.32)	0.88 (0.80)
Product issues	459	2.69 (2.43–2.97) *	2.49 (414.47) *	1.28 (1.13) *	2.44 (2.21) *
Psychiatric disorders	403	0.72 (0.65–0.8)	0.75 (39.53)	−0.42 (−0.57)	0.75 (0.68)
Eye disorders	306	2.08 (1.84–2.34) *	1.99 (152.83)	0.96 (0.79) *	1.96 (1.74)
Metabolism and nutrition disorders	263	0.85 (0.75–0.97)	0.86 (6.30)	−0.22 (−0.4)	0.86 (0.76)
Surgical and medical procedures	251	2.92 (2.56–3.32) *	2.79 (284.79) *	1.43 (1.24) *	2.73 (2.39) *
Cardiac disorders	245	0.74 (0.65–0.85)	0.76 (19.82)	−0.39 (−0.58)	0.76 (0.67)
Blood and lymphatic system disorders	209	0.69 (0.60–0.80)	0.71 (26.24)	−0.49 (−0.70)	0.71 (0.62)
Pregnancy, puerperium, and perinatal conditions	207	1.13 (0.98–1.30)	1.12 (2.83)	0.16 (−0.05)	1.12 (0.97)
Neoplasms benign, malignant, and unspecified (incl. cysts and polyps)	152	1.44 (1.22–1.70) *	1.42 (19.39)	0.49 (0.25) *	1.42 (1.20)
Renal and urinary disorders	133	0.80 (0.67–0.95)	0.81 (6.37)	−0.32 (−0.57)	0.81 (0.68)
Reproductive system and breast disorders	95	0.73 (0.59–0.89)	0.73 (9.48)	−0.45 (−0.76)	0.74 (0.60)
Hepatobiliary disorders	79	0.68 (0.54–0.85)	0.68 (11.87)	−0.56 (−0.89)	0.69 (0.55)
Congenital, familial, and genetic disorders	71	0.73 (0.58–0.92)	0.73 (6.93)	−0.46 (−0.80)	0.74 (0.58)
Social circumstances	67	2.85 (2.23–3.64) *	2.81 (75.98) *	1.40 (1.04) *	2.75 (2.15) *
Endocrine disorders	56	0.35 (0.27–0.45)	0.36 (67.43)	−1.48 (−1.87)	0.36 (0.28)
Ear and labyrinth disorders	54	1.31 (1.00–1.72)	1.31 (3.85)	0.34 (−0.05) *	1.30 (0.99)

* indicates statistically significant signals in algorithm. ROR, reporting odds ratio; CI, confidence interval; PRR, proportional reporting ratio; χ^2^, chi-squared; IC, information component; IC025, the lower limit of 95% CI of the IC; EBGM, empirical Bayesian geometric mean; EBGM05, the lower limit of 95% CI of EBGM.

**Table 3:** Signal strength of reports of adalimumab (in children) at the Preferred Term (PT) level in FAERS database.

SOC	Preferred Terms (PTs)	Reports	ROR (95% Two-Sided CI)	PRR (χ^2^)	IC (IC025)	EBGM (EBGM05)
Blood and lymphatic system disorders	Lymphadenitis	14	10.16 (5.80–17.79)	10.13 (101.19)	2.46 (1.64)	9.02 (5.15)
Eye disorders	Uveitis	75	36.28 (27.48–47.89)	35.58 (1696.6)	4.20 (3.81)	24.26 (18.37)
	Visual impairment	35	3.58 (2.55–5.03)	3.56 (61.50)	1.65 (1.15)	3.44 (2.45)
	Cataract	29	11.35 (7.67–16.80)	11.27 (235.32)	2.88 (2.31)	9.90 (6.69)
	Eye inflammation	23	21.39 (13.43–34.08)	21.27 (344.26)	3.27 (2.61)	16.70 (10.49)
	Glaucoma *	22	22.76 (14.10–36.76)	22.64 (347.51)	3.29 (2.60)	17.52 (10.85)
	Eye disorder	19	5.58 (3.49–8.90)	5.55 (65.98)	2.04 (1.35)	5.23 (3.28)
	Blindness	18	4.77 (2.96–7.68)	4.75 (50.05)	1.85 (1.15)	4.52 (2.80)
	Blindness unilateral	17	34.65 (19.45–61.75)	34.5 (375.71)	3.31 (2.50)	23.76 (13.33)
	Iridocyclitis	17	21.14 (12.31–36.30)	21.05 (252.13)	3.07 (2.29)	16.57 (9.65)
Gastrointestinal disorders	Crohn’s disease	305	16.55 (14.56–18.80)	15.31 (3392.83)	3.62 (3.44)	12.83 (11.29)
	Abdominal pain	162	3.06 (2.61–3.59)	2.97 (206.67)	1.51 (1.27)	2.89 (2.47)
	Hematochezia	118	7.43 (6.13–9.00)	7.23 (579.38)	2.66 (2.38)	6.67 (5.51)
	Intestinal obstruction *	60	19.78 (14.85–26.34)	19.48 (831.44)	3.63 (3.22)	15.59 (11.71)
	Colitis ulcerative	53	3.89 (2.94–5.13)	3.85 (106.41)	1.79 (1.38)	3.70 (2.80)
	Colitis	52	8.40 (6.29–11.21)	8.30 (300.15)	2.72 (2.30)	7.55 (5.66)
	Gastrointestinal inflammation	42	31.33 (21.81–45.01)	31.00 (856.47)	3.85 (3.35)	22.06 (15.36)
	Anal fistula	38	53.92 (35.44–82.02)	53.39 (1129.12)	4.10 (3.54)	31.27 (20.56)
	Frequent bowel movements	37	8.14 (5.79–11.45)	8.07 (206.64)	2.62 (2.12)	7.37 (5.24)
	Diarrhea hemorrhagic	25	7.92 (5.24–11.98)	7.87 (135.54)	2.48 (1.88)	7.20 (4.76)
	Intestinal stenosis *	25	79.93 (45.33–140.95)	79.42 (927.70)	3.92 (3.21)	38.57 (21.88)
	Ileal stenosis *	24	70.58 (40.27–123.69)	70.14 (834.7)	3.85 (3.13)	36.28 (20.7)
	Rectal hemorrhage	24	6.81 (4.47–10.35)	6.77 (108.13)	2.32 (1.70)	6.28 (4.13)
	Small intestinal obstruction *	21	16.41 (10.21–26.36)	16.32 (247)	3.04 (2.35)	13.52 (8.42)
	Gastritis	20	5.54 (3.51–8.73)	5.51 (68.79)	2.04 (1.38)	5.20 (3.30)
	Large intestinal stenosis *	16	61.78 (31.75–120.23)	61.53 (517.24)	3.44 (2.57)	33.86 (17.40)
	Intestinal perforation	15	9.32 (5.44–15.97)	9.29 (98.47)	2.42 (1.64)	8.35 (4.88)
	Gastrointestinal pain	10	10.61 (5.46–20.62)	10.59 (75.86)	2.27 (1.31)	9.38 (4.83)
	Intestinal fistula	10	122.09 (44.35–336.1)	121.77 (449.20)	3.04 (1.88)	46.29 (16.82)
General disorders and administration site conditions	Injection site pain	288	7.99 (7.05–9.07)	7.47 (1479.77)	2.75 (2.56)	6.87 (6.06)
	Injection site hemorrhage	271	46.15 (39.58–53.82)	42.95 (7011.17)	4.64 (4.43)	27.42 (23.51)
	Pain	120	3.05 (2.54–3.68)	2.99 (154.38)	1.51 (1.24)	2.91 (2.42)
	Injection site erythema	77	8.47 (6.68–10.75)	8.32 (446.53)	2.79 (2.44)	7.57 (5.97)
	Asthenia	66	2.83 (2.20–3.62)	2.79 (73.68)	1.39 (1.02)	2.73 (2.13)
	Inflammation	63	10.48 (8.03–13.67)	10.32 (465.50)	3.00 (2.61)	9.17 (7.03)
	Injection site reaction	59	10.34 (7.86–13.60)	10.19 (429.92)	2.97 (2.57)	9.07 (6.89)
	Therapeutic product effect incomplete	55	6.56 (4.97–8.66)	6.48 (234.75)	2.44 (2.04)	6.03 (4.57)
	Injection site swelling	41	5.00 (3.64–6.88)	4.96 (121.65)	2.08 (1.61)	4.71(3.43)
	Therapeutic product effect decreased	41	6.34 (4.60–8.73)	6.28 (167.91)	2.36 (1.89)	5.86 (4.25)
	Injection site papule	32	261.97 (124.96–549.19)	259.78 (1811.04)	4.36 (3.68)	57.81 (27.57)
	Injection site bruising	31	4.03 (2.81–5.80)	4.01 (66.50)	1.78 (1.24)	3.85 (2.68)
	Illness	26	3.19 (2.15–4.72)	3.17 (37.13)	1.46 (0.88)	3.08 (2.08)
	Injection site injury	21	61.71 (34.51–110.33)	61.37 (677.93)	3.70 (2.94)	33.81 (18.91)
	Injection site pruritus	21	5.71 (3.66–8.91)	5.68 (75.26)	2.09 (1.44)	5.34 (3.42)
	Injection site urticaria	20	5.87 (3.72–9.27)	5.85 (74.44)	2.11 (1.44)	5.49 (3.48)
	Gait inability	19	5.22 (3.27–8.32)	5.20 (60.22)	1.97 (1.28)	4.92 (3.09)
	Impaired healing	16	9.78 (5.8–16.49)	9.74 (110.8)	2.50 (1.73)	8.71 (5.17)
	Therapeutic response shortened	15	5.47 (3.23–9.25)	5.45 (50.79)	1.94 (1.17)	5.14 (3.04)
	Injection site vesicles	14	15.79 (8.86–28.16)	15.74 (159.01)	2.76 (1.92)	13.13 (7.36)
	Injection site rash	13	5.09 (2.90–8.94)	5.08 (39.85)	1.81 (0.99)	4.81 (2.74)
	Injection site indentation	12	97.72 (41.15–232.06)	97.42 (490.81)	3.22 (2.18)	42.32 (17.82)
	Injury associated with device	12	9.25 (5.07–16.89)	9.23 (78.2)	2.30 (1.42)	8.31 (4.55)
	Mass	12	8.71 (4.78–15.85)	8.68 (72.92)	2.25 (1.37)	7.87 (4.32)
	Cyst	11	9.16 (4.89–17.15)	9.13 (70.84)	2.23 (1.32)	8.23 (4.39)
Immune system disorders	Immunodeficiency *	17	8.97 (5.42–14.86)	8.94 (106.81)	2.45 (1.71)	8.07 (4.87)
Infections and infestations	Anal abscess *	18	11.49 (6.98–18.9)	11.44 (148.29)	2.69 (1.96)	10.02 (6.09)
	Pharyngitis streptococcal	15	3.60 (2.14–6.06)	3.59 (26.79)	1.50 (0.73)	3.47 (2.07)
	Tonsillitis	13	4.31 (2.46–7.55)	4.30 (31.09)	1.64 (0.82)	4.11 (2.35)
	Abdominal abscess *	12	19.12 (10.12–36.12)	19.06 (162.90)	2.75 (1.84)	15.32 (8.11)
	Gastrointestinal infection	12	6.42 (3.55–11.59)	6.40 (50.30)	1.99 (1.13)	5.96 (3.30)
	Abscess *	11	4.91 (2.67–9.05)	4.90 (32.02)	1.71 (0.81)	4.66 (2.53)
	Disseminated tuberculosis	11	25.19 (12.69–50)	25.12 (189.58)	2.80 (1.82)	18.95 (9.54)
	Rhinitis	10	4.09 (2.16–7.74)	4.08 (22.05)	1.49 (0.56)	3.92 (2.07)
Injury, poisoning, and procedural complications	Incorrect dose administered	371	10.95 (9.77–12.26)	9.98 (2666.68)	3.12 (2.95)	8.90 (7.95)
	Wrong technique in product usage process	310	12.39 (10.94–14.03)	11.47 (2581.15)	3.28 (3.10)	10.05 (8.87)
	Exposure via breast milk	42	4.19 (3.07–5.73)	4.16 (95.58)	1.86 (1.41)	3.99(2.92)
	Maternal exposure during breast feeding	17	9.90 (5.96–16.44)	9.86 (119.28)	2.54 (1.80)	8.80 (5.30)
	Road traffic accident	12	6.28 (3.48–11.33)	6.26 (48.9)	1.98 (1.11)	5.85 (3.24)
	Post procedural complication	11	6.71 (3.62–12.46)	6.70 (48.86)	1.99 (1.08)	6.22 (3.35)
	Procedural pain	11	3.97 (2.16–7.28)	3.96 (23.1)	1.50 (0.61)	3.81 (2.07)
	Paternal drugs affecting fetus	10	366.27 (80.22–1672.25)	365.32 (605.56)	3.11 (1.87)	61.72 (13.52)
Investigations	Weight decreased *	90	3.67 (2.96–4.55)	3.61 (162.86)	1.75 (1.43)	3.49 (2.81)
	Drug level decreased	41	9.25 (6.67–12.82)	9.16 (265.21)	2.78 (2.30)	8.25 (5.95)
	C-reactive protein increased *	38	4.24 (3.05–5.89)	4.21(88.05)	1.87 (1.38)	4.03 (2.90)
	Hemoglobin decreased	38	3.47 (2.50–4.82)	3.45 (63.29)	1.62 (1.14)	3.34 (2.41)
	Drug specific antibody present	28	13.04 (8.71–19.51)	12.95 (262.41)	3.00 (2.41)	11.15 (7.45)
	Inflammatory marker increased	25	15.57 (10.10–24.01)	15.48 (279.55)	3.09 (2.47)	12.95 (8.40)
	Intraocular pressure increased *	23	13.41 (8.59–20.94)	13.34 (222.08)	2.93 (2.29)	11.43 (7.32)
	White blood cell count increased	22	4.28 (2.78–6.59)	4.26 (52.00)	1.78 (1.15)	4.08 (2.65)
	Blood iron decreased *	21	22.03 (13.52–35.92)	21.92 (322.61)	3.24 (2.54)	17.09 (10.48)
	Red blood cell sedimentation rate increased	19	7.83 (4.88–12.58)	7.80 (101.82)	2.38 (1.68)	7.14 (4.45)
	Fecal calprotectin increased *	18	29.36 (16.98–50.76)	29.23 (350.56)	3.28 (2.51)	21.16 (12.24)
	Fetal heart rate decreased *	14	38.02 (19.92–72.56)	37.88 (331.13)	3.17 (2.28)	25.29 (13.25)
Metabolism and nutrition disorders	Hypophagia *	26	4.69 (3.15–6.98)	4.67 (70.53)	1.93 (1.34)	4.45 (2.99)
	Malnutrition *	24	8.56 (5.60–13.08)	8.51 (142.63)	2.55 (1.93)	7.73 (5.06)
Musculoskeletal and connective tissue disorders	Arthralgia	96	3.45 (2.80–4.24)	3.39 (155.65)	1.67 (1.36)	3.28 (2.67)
	Juvenile idiopathic arthritis	70	9.94 (7.73–12.79)	9.78 (487.59)	2.96 (2.59)	8.74 (6.80)
	Arthritis	44	8.87 (6.48–12.15)	8.78 (271.31)	2.75 (2.29)	7.95 (5.80)
	Joint swelling	37	5.56(3.98–7.78)	5.52 (127.47)	2.19 (1.70)	5.20 (3.72)
	Mobility decreased	23	5.38 (3.52–8.22)	5.35 (75.92)	2.05 (1.43)	5.05 (3.31)
	Arthropathy	21	5.91 (3.78–9.22)	5.88 (78.77)	2.13 (1.47)	5.52 (3.53)
	Rheumatoid arthritis	20	6.99 (4.41–11.07)	6.96 (93.25)	2.28 (1.61)	6.44 (4.07)
	Fistula	19	14.84 (9.05–24.32)	14.77 (202.96)	2.91 (2.19)	12.45 (7.60)
	Musculoskeletal stiffness	18	3.75 (2.33–6.03)	3.74 (34.36)	1.59 (0.89)	3.60 (2.24)
	Systemic lupus erythematosus	18	11.59 (7.04–19.07)	11.54 (149.66)	2.69 (1.97)	10.10 (6.14)
	Joint effusion	16	9.17 (5.45–15.43)	9.13 (103.06)	2.44 (1.68)	8.23 (4.89)
	Chronic recurrent multifocal osteomyelitis	14	46.66 (23.86–91.27)	46.49 (380.91)	3.24 (2.33)	28.80 (14.73)
	Synovitis	11	7.82 (4.20–14.58)	7.80 (58.97)	2.12 (1.20)	7.15 (3.83)
	Osteoarthritis	10	9.39 (4.86–18.15)	9.37 (66.27)	2.19 (1.23)	8.42 (4.35)
Neoplasms benign, malignant, and unspecified (incl. cysts and polyps)	Lymphoma	13	14.44 (7.96–26.19)	14.39 (135.37)	2.65 (1.79)	12.19 (6.72)
Product issues	Device issue	384	17.20 (15.33–19.30)	15.57 (4349.32)	3.65 (3.49)	13.01 (11.59)
	Needle issue	28	4.07 (2.78–5.97)	4.05 (61.08)	1.77 (1.21)	3.89 (2.66)
Psychiatric disorders	Fear of injection	43	18.36 (13.12–25.67)	18.16 (558.88)	3.46 (2.97)	14.74 (10.54)
	Depressed mood	36	3.32 (2.37–4.64)	3.3 (55.26)	1.55 (1.06)	3.20 (2.29)
	Fear	26	6.92 (4.63–10.36)	6.88 (119.60)	2.36 (1.77)	6.38 (4.26)
	Stress	17	4.45 (2.73–7.27)	4.44 (42.70)	1.76 (1.04)	4.24 (2.60)
Renal and urinary disorders	IgA nephropathy	10	45.78 (20.76–100.95)	45.66 (268.86)	2.89 (1.82)	28.49 (12.92)
Respiratory, thoracic, and mediastinal disorders	Tonsillar hypertrophy	10	6.91 (3.61–13.22)	6.89 (46.05)	1.96 (1.01)	6.38 (3.34)
Skin and subcutaneous tissue disorders	Psoriasis	60	11.09 (8.44–14.58)	10.93 (471.72)	3.05 (2.66)	9.64 (7.34)
	Hidradenitis	18	50.82 (27.84–92.76)	50.58 (517.03)	3.50 (2.69)	30.30 (16.6)
Social circumstances	Loss of personal independence in daily activities	45	11.79 (8.59–16.17)	11.66 (378.61)	3.05 (2.59)	10.19 (7.43)
Vascular disorders	Takayasu’s arteritis *	11	36.63 (17.75–75.60)	36.53 (253.46)	2.93 (1.92)	24.69 (11.96)

* Emerging findings of adalimumab-associated AEs from FAERS database, and emerging findings refer to PTs that are not explicitly listed in the drug package insert. ROR, reporting odds ratio; CI, confidence interval; PRR, proportional reporting ratio; χ^2^, chi-squared; IC, information component; IC025, the lower limit of 95% CI of the IC; EBGM, empirical Bayesian geometric mean; EBGM05, the lower limit of 95% CI of EBGM.

**Table 4:** The results of time-to-onset analysis for signals with different populations.

Group	TTO (Days)			Weibull Distribution				Failure Type
	Cases			Scale Parameter		Shape Parameter		
	n	Median (IQR)	Min–Max	α	95% CI	β	95% CI	
Children	1823	99 (14–336)	0–1279	115.78	102.09–131.31	0.38	0.36–0.39	Early failure
All	40,126	124 (29–301)	0–1299	163.48	159.58–167.48	0.42	0.41–0.42	Early failure

n, number of cases with available time-to-onset; IQR, interquartile range; TTO, time-to-onset. When TTO is 0 days, the adverse event occurred within the same day with the therapy.

## Data Availability

All the data generated or analyzed during this study are included in this published article and its Supplementary Files. The database used in this study is publicly available at the website of URL (https://fis.fda.gov/extensions/FPD-QDE-FAERS/FPD-QDE-FAERS.html, accessed on 3 February 2023).

## Supplementary Materials

The following supporting information can be downloaded at: https://www.mdpi.com/article/10.3390/ph17081028/s1, Table S1. Clinical characteristics of reports with adalimumab from the FAERS database (January 2017 to September 2022). Table S2. Signal strength of reports of adalimumab (in children) at the Preferred Term (PT) level in FAERS database. Table S3. Summary of FDA-approved adalimumab. Table S4. Four major algorithms used for signal detection.
