# Supplementary material for: Adverse Event Profiles of Adalimumab in Children: A Disproportionality Analysis
Source: Pharmaceuticals (Basel). 2024 Aug 5;17(8):1028. doi: 10.3390/ph17081028 (PMC11357133; doi:10.3390/ph17081028)
Supplement: Supplementary file 1 [file pharmaceuticals-17-01028-s001.zip › pharmaceuticals-3068359-supplementary.pdf]

Supplementary Table S1. Clinical characteristics of reports with adalimumab from the FAERS database (January 2017 to September 2022).

Characteristics	Adalimumab induced AEs reports (n=192,144)		
Number of events	Available number, n	Case number, n	Case proportion, %
Gender, n (%)	182,494	-	94.98
Female	-	117,376	64.32
Male	-	65,118	35.68
Age (years), n (%)	102,371	-	53.28
<18	-	3819	3.73
18≤and≤65	-	73,537	71.83
>65	-	25,015	24.44
Median (IQR)	-	55 (40-65)	-
Weight (Kg), n (%)	46,791	-	24.35
<80	-	26,134	55.85
80≤and≤100	-	13,036	27.86
>100	-	7621	16.29
Median (IQR)	-	77 (63.56-92.16)	-
Reported countries, n (%)	178,329	-	92.81
US	-	123,601	69.31
Non-US	-	54,728	30.69
Indications, n (%)	171,192	-	89.10
Rheumatoid arthritis	-	51,923	30.33
Crohn's disease		41,182	24.06
Psoriasis		20,787	12.14
Psoriatic arthropathy		15,789	9.22
Colitis ulcerative		12,776	7.46
Ankylosing spondylitis		10,450	6.10
Hidradenitis		8478	4.95
Uveitis		2153	1.26
Juvenile idiopathic arthritis		1609	0.94
Arthritis		443	0.26
Outcomes, n (%)	192,144	-	100.00
Non-serious Outcome	-	61,879	32.20
Serious outcome[a]	-	130,265	67.80
Death	-	10,007	7.68
Life-threatening	-	1398	1.07
Hospitalization	-	54,847	42.10
Disability	-	2840	2.18
Other serious outcomes	-	87,548	67.21
Time-to-onset (days)	40,126	-	20.88

Median (IQR)	-	149 (28-434)	-
Reporters, n (%)	187,921	-	97.80
Health professional	-	51,998	27.67
Consumer	-	135,923	72.33
Reporting year, n (%)	192,144	-	100.00
2022 Q3[b]	-	29,597	15.40
2021	-	32,630	16.98
2020	-	35,576	18.52
2019	-	34,460	17.93
2018		31,144	16.21
2017		28,737	14.96

a, Total serious outcomes may exceed the total number of reported cases because some cases list more than one serious outcomes.

b, The third quarter of 2022.

IQR, interquartile range

Supplementary Table S2. Signal strength of reports of adalimumab (in children) at the Preferred Term (PT) level in FAERS database.

SOC	Preferred Terms (PTs)	Reports	ROR (95% two-sided CI)	PRR (χ2)	IC (IC025)	EBGM (EBGM05)
Blood and lymphatic system disorders	Increased tendency to bruise	5	14.07 (5.40-36.65)	14.05 (50.84)	1.82 (0.43)	11.95 (4.58)
Cardiac disorders	Foetal heart rate deceleration abnormality	6	6.10(2.65-14.03)	6.09 (23.56)	1.55 (0.32)	5.70 (2.48)
Congenital, familial and genetic disorders	Blau syndrome	7	512.38 (63.02-4165.7)	511.44 (445.78)	2.66 (1.17)	64.81 (7.97)
Eye disorders	Iris adhesions	6	19.09 (7.77-46.91)	19.06 (81.44)	2.11 (0.81)	15.32 (6.24)
	Macular oedema	5	11.80 (4.59-30.36)	11.78 (42.50)	1.75 (0.37)	10.29 (4.00)
	Retinal detachment	5	6.77 (2.71-16.94)	6.77 (22.49)	1.48 (0.13)	6.28 (2.51)
	Anterior chamber cell	4	22.50 (7.33-69.05)	22.48 (62.79)	1.70 (0.10)	17.43 (5.68)
	Iritis	4	17.21 (5.79-51.17)	17.19 (49.38)	1.64 (0.07)	14.11 (4.74)
	Keratopathy	4	22.50 (7.33-69.05)	22.48 (62.79)	1.70 (0.10)	17.43 (5.68)
	Scleritis	4	32.51 (10.01-105.6)	32.47 (84.47)	1.77 (0.12)	22.79 (7.01)
Gastrointestinal disorders	Enteritis	9	5.36 (2.72-10.55)	5.35 (29.64)	1.69 (0.70)	5.05 (2.56)
	Abdominal adhesions	8	58.57 (23.10-148.49)	58.45 (250.98)	2.69 (1.46)	32.92 (12.98)
	Abnormal faeces	8	4.68 (2.29-9.58)	4.68 (21.74)	1.52 (0.47)	4.45 (2.18)
	Defaecation urgency	7	17.08 (7.50-38.9)	17.05 (85.75)	2.22 (1.03)	14.01 (6.15)
	Gastrointestinal obstruction	7	30.14 (12.49-72.72)	30.08 (139.43)	2.40 (1.16)	21.60 (8.95)
	Ileal ulcer	7	32.02 (13.17-77.88)	31.97 (146.08)	2.42 (1.17)	22.54 (9.27)
	Intestinal haemorrhage	7	9.49 (4.31-20.86)	9.47 (46.96)	1.94 (0.79)	8.50 (3.86)
	Faeces soft	6	6.18 (2.69-14.23)	6.17 (24.00)	1.56 (0.33)	5.77 (2.51)
	Gastrointestinal oedema	6	36.59 (13.72-97.54)	36.53 (138.25)	2.27 (0.91)	24.69 (9.26)
	Irritable bowel syndrome	6	11.26 (4.76-26.6)	11.24 (48.52)	1.90 (0.65)	9.88 (4.18)
	Proctalgia	6	7.32 (3.16-16.94)	7.31 (29.70)	1.67 (0.44)	6.73 (2.91)

	Anal fissure	5	7.95 (3.16-20.02)	7.94 (27.37)	1.57 (0.21)	7.26 (2.88)
	Functional gastrointestinal disorder	5	12.19 (4.73-31.44)	12.18 (43.97)	1.76 (0.38)	10.58 (4.10)
	Gastrointestinal fistula	5	365.79 (42.72-3131.86)	365.32 (302.77)	2.21 (0.47)	61.72 (7.21)
	Gastrointestinal stenosis	5	365.79 (42.72-3131.86)	365.32 (302.77)	2.21 (0.47)	61.72 (7.21)
	Large intestine perforation	5	6.77 (2.71-16.94)	6.77 (22.49)	1.48 (0.13)	6.28 (2.51)
	Small intestinal stenosis	5	121.93 (29.13-510.4)	121.77 (224.59)	2.17 (0.53)	46.29 (11.06)
	Anal haemorrhage	4	14.63 (5.00-42.81)	14.61 (42.27)	1.60 (0.04)	12.34 (4.22)
	Intestinal ulcer	4	22.50 (7.33-69.05)	22.48 (62.79)	1.70 (0.10)	17.43 (5.68)
	Rectal discharge	4	41.79 (12.23-142.83)	41.75 (101.25)	1.80 (0.12)	26.93 (7.88)
General disorders and administration site conditions	Obstruction	7	19.71 (8.55-45.43)	19.67 (97.75)	2.28 (1.08)	15.71 (6.81)
	Granuloma	5	5.80 (2.33-14.44)	5.80 (18.4)	1.38 (0.05)	5.45 (2.19)
	Ulcer	5	5.80 (2.33-14.44)	5.80 (18.4)	1.38 (0.05)	5.45 (2.19)
Hepatobiliary disorders	Autoimmune hepatitis	7	5.69 (2.64-12.29)	5.68 (25.07)	1.60 (0.47)	5.34 (2.48)
	Cholangitis sclerosing	6	7.98 (3.43-18.55)	7.97 (32.99)	1.72 (0.48)	7.28 (3.13)
Immune system disorders	Immunosuppression	8	5.80 (2.82-11.92)	5.79 (29.37)	1.69 (0.64)	5.44 (2.64)
	Seasonal allergy	6	5.05 (2.20-11.55)	5.04 (18.18)	1.41 (0.20)	4.78 (2.09)
Infections and infestations	Rash pustular	9	7.66 (3.85-15.24)	7.65 (47.08)	1.98 (0.97)	7.02 (3.53)
	Abscess intestinal	8	58.57 (23.10-148.49)	58.45 (250.98)	2.69 (1.46)	32.92 (12.98)
	Localised infection	7	5.23 (2.43-11.26)	5.22 (22.29)	1.53 (0.41)	4.94 (2.29)
	Post procedural infection	7	13.14 (5.87-29.39)	13.11 (66.42)	2.11 (0.94)	11.27 (5.04)
	Subcutaneous abscess	7	6.92 (3.19-15.04)	6.91 (32.34)	1.74 (0.60)	6.40 (2.95)
	Postoperative wound infection	6	8.61 (3.69-20.07)	8.60 (36.04)	1.76 (0.52)	7.80 (3.34)
	Abscess limb	5	6.77 (2.71-16.94)	6.77 (22.49)	1.48 (0.13)	6.28 (2.51)
	Folliculitis	5	5.63 (2.26-13.98)	5.62 (17.64)	1.36 (0.03)	5.29 (2.13)

	Gastric infection	5	9.14 (3.61-23.18)	9.13 (32.19)	1.64 (0.28)	8.23 (3.25)
	Purulence	5	40.64 (13.61-121.33)	40.59 (124.13)	2.07 (0.57)	26.45 (8.86)
	Purulent discharge	5	7.78 (3.09-19.58)	7.77 (26.67)	1.55 (0.20)	7.12 (2.83)
	Rectal abscess	5	21.52 (7.93-58.35)	21.49 (75.48)	1.95 (0.52)	16.83 (6.21)
Injury, poisoning and procedural complications	Wound	8	4.76 (2.33-9.74)	4.75 (22.26)	1.53 (0.48)	4.52 (2.21)
	Gastrointestinal stoma complication	7	24.40 (10.37-57.43)	24.35 (117.59)	2.34 (1.13)	18.52 (7.87)
	Product administered at inappropriate site	7	6.25 (2.89-13.52)	6.24 (28.37)	1.67 (0.54)	5.83 (2.69)
	Arthropod bite	6	9.98 (4.25-23.43)	9.96 (42.58)	1.84 (0.59)	8.89 (3.79)
	Paternal exposure during pregnancy	5	45.72 (14.95-139.83)	45.66 (134.43)	2.09 (0.57)	28.49 (9.31)
	Meniscus injury	4	32.51 (10.01-105.60)	32.47 (84.47)	1.77 (0.12)	22.79 (7.01)
Investigations	Drug specific antibody	8	6.97 (3.37-14.41)	6.96 (37.28)	1.84 (0.77)	6.44 (3.12)
	Neutrophil count increased	8	4.80 (2.35-9.82)	4.79 (22.53)	1.54 (0.49)	4.56 (2.23)
	Full blood count abnormal	6	17.56 (7.20-42.83)	17.54 (75.45)	2.08 (0.79)	14.33 (5.88)
	Antinuclear antibody positive	5	8.71 (3.44-22.02)	8.70 (30.45)	1.61 (0.26)	7.88 (3.12)
	Mean platelet volume decreased	5	6.53 (2.61-16.31)	6.52 (21.47)	1.46 (0.11)	6.07 (2.43)
	Red blood cell sedimentation rate abnormal	5	7.62 (3.03-19.15)	7.61 (26.01)	1.54 (0.19)	6.99 (2.78)
	Drug clearance increased	4	41.79 (12.23-142.83)	41.75 (101.25)	1.80 (0.12)	26.93 (7.88)
	Endoscopy	4	48.76 (13.75-172.86)	48.71 (112.15)	1.82 (0.11)	29.63 (8.36)
	Nutritional condition abnormal	4	73.14 (18.28-292.56)	73.06 (142.16)	1.85 (0.09)	37.03 (9.26)
Metabolism and nutrition disorders	Iron deficiency	6	8.96 (3.84-20.93)	8.95 (37.74)	1.78 (0.54)	8.08 (3.46)
Musculoskeletal and connective tissue disorders	Joint destruction	7	6.17 (2.85-13.36)	6.16 (27.92)	1.66 (0.53)	5.76 (2.66)
	Joint range of motion decreased	7	12.81 (5.73-28.61)	12.79 (64.73)	2.10 (0.93)	11.03 (4.94)
	Polyarthritis	7	4.70 (2.19-10.10)	4.69 (19.12)	1.45 (0.32)	4.47 (2.08)
	Psoriatic arthropathy	6	25.83 (10.18-65.54)	25.79 (105.67)	2.19 (0.87)	19.32 (7.61)
	Sacroiliitis	6	12.19 (5.14-28.96)	12.18 (52.77)	1.94 (0.68)	10.58 (4.46)

	SAPHO syndrome	6	17.56 (7.20-42.83)	17.54 (75.45)	2.08 (0.79)	14.33 (5.88)
	Rheumatoid nodule	5	182.9 (35.47-943.02)	182.66 (258.10)	2.19 (0.51)	52.90 (10.26)
	Spinal pain	5	9.89 (3.88-25.17)	9.87 (35.13)	1.67 (0.31)	8.82 (3.46)
	Ankylosing spondylitis	4	17.21 (5.79-51.17)	17.19 (49.38)	1.64 (0.07)	14.11 (4.74)
	Rheumatic disorder	4	36.57 (11.01-121.5)	36.53 (92.16)	1.78 (0.12)	24.69 (7.43)
	Spondylitis	4	97.52 (21.82-435.88)	97.42 (163.60)	1.87 (0.07)	42.32 (9.47)
Neoplasms benign, malignant and unspecified (incl cysts and polyps)	Leukaemia	6	6.27 (2.72-14.44)	6.26 (24.45)	1.57 (0.34)	5.85 (2.54)
Nervous system disorders	Demyelination	5	6.42 (2.57-16.02)	6.41 (20.99)	1.44 (0.10)	5.97 (2.39)
Pregnancy, puerperium and perinatal conditions	Breech presentation	7	17.67 (7.73-40.35)	17.64 (88.50)	2.24 (1.04)	14.40 (6.30)
	Umbilical cord around neck	7	15.07 (6.68-34.01)	15.04 (76.11)	2.17 (0.99)	12.64 (5.60)
Renal and urinary disorders	Renal colic	4	22.50 (7.33-69.05)	22.48 (62.79)	1.70 (0.10)	17.43 (5.68)
Reproductive system and breast disorders	Haemorrhagic ovarian cyst	4	20.90 (6.87-63.51)	20.88 (58.88)	1.69 (0.09)	16.46 (5.41)
Respiratory, thoracic and mediastinal disorders	Meconium aspiration syndrome	5	7.78 (3.09-19.58)	7.77 (26.67)	1.55 (0.20)	7.12 (2.83)
	Neonatal aspiration	5	30.48 (10.73-86.57)	30.44 (100.51)	2.02 (0.56)	21.78 (7.67)
Skin and subcutaneous tissue disorders	Pyoderma gangrenosum	8	27.89 (12.35-63.01)	27.83 (149.88)	2.52 (1.37)	20.43 (9.04)
	Skin mass	7	6.03 (2.79-13.03)	6.02 (27.06)	1.64 (0.51)	5.64 (2.61)
	Erythema nodosum	6	11.26 (4.76-26.60)	11.24 (48.52)	1.90 (0.65)	9.88 (4.18)
Vascular disorders	Vasculitis	8	6.04 (2.93-12.42)	6.03 (30.99)	1.73 (0.67)	5.64 (2.74)
	Behcet's syndrome	5	13.06 (5.04-33.85)	13.05 (47.20)	1.79 (0.41)	11.22 (4.33)
	Neurogenic shock	4	14.63 (5.00-42.81)	14.61 (42.27)	1.60 (0.04)	12.34 (4.22)

ROR, reporting odds ratio; CI, confidence interval; PRR, proportional reporting ratio; χ^2, chi-squared; IC, information component; IC025, the lower limit of 95% CI of the IC; EBGM, empirical Bayesian geometric mean; EBGM05, the lower limit of 95% CI of EBGM.

Supplementary Table S3. Summary of FDA-approved adalimumab.

Target	Brand name	Generic name	Manufacturer	Approval date
TNF-α	ABRILADA	ADALIMUMAB-AFZB	PFIZER INC	11/15/2019
	AMJEVITA	ADALIMUMAB-ATTO	AMGEN INC	09/23/2016
	CYLTEZO	ADALIMUMAB-ADBM	BOEHRINGER INGELHEIM	08/25/2017
	HADLIMA	ADALIMUMAB-BWWD	SAMSUNG BIOEPIS CO LTD	07/23/2019
	HULIO	ADALIMUMAB-FKJP	MYLAN PHARMS INC	07/06/2020
	HUMIRA	ADALIMUMAB	ABBVIE INC	12/31/2002
	HYRIMOZ	ADALIMUMAB-ADAZ	SANDOZ INC	10/30/2018
	IDACIO	ADALIMUMAB-AACF	FRESENIUS KABI USA	12/13/2022
	YUSIMRY	ADALIMUMAB-AQVH	COHERUS BIOSCIENCES INC	12/17/2021

Supplementary Table S4. Four major algorithms used for signal detection.

Algorithms	Equation	Criteria
ROR	ROR=(a/c)/(b/d)<br>95%CI=$e^{\ln(ROR)\pm 1.96\sqrt{\left(\frac{1}{a}+\frac{1}{b}+\frac{1}{c}+\frac{1}{d}\right)}}$	lower limit of 95% CI>1, N≥3
PRR	PRR=a(c+d)/c/(a+b)<br>χ^2=[(ad-bc)^2](a+b+c+d)/[(a+b)(c+d)(a+c)(b+d)]	PRR≥2, χ^2≥4, N≥3
BCPNN	IC=$\log_2$a(a+b+c+d)(a+c)(a+b)<br>95%CI= E(IC) ± $2\sqrt{V(IC)}$	IC025>0
MGPS	EBGM=a(a+b+c+d)/(a+c)/(a+b)<br>95%CI=$e^{\ln(EBGM)\pm 1.96\sqrt{\left(\frac{1}{a}+\frac{1}{b}+\frac{1}{c}+\frac{1}{d}\right)}}$	EBGM05>2

Equation: a, number of reports containing both the target drug and target adverse drug reaction; b, number of reports containing other adverse drug reaction of the target drug; c, number of reports containing the target adverse drug reaction of other drugs; d, number of reports containing other drugs and other adverse drug reactions. 95%CI, 95% confidence interval; N, the number of reports; χ^2, chi-squared; IC, information component; IC025, the lower limit of 95% CI of the IC; E(IC), the IC expectations; V(IC), the variance of IC; EBGM, empirical Bayesian geometric mean; EBGM05, the lower limit of 95% CI of EBGM.
